# Thermodynamics of Sulfur Vacancy Formation in the Chalcogenide Perovskite BaZrS_3_

**DOI:** 10.1021/acs.jpcc.5c00828

**Published:** 2025-10-22

**Authors:** Zhenzhu Li, Aron Walsh

**Affiliations:** † Department of Materials, 4615 Imperial College London, London SW7 2AZ, U.K.; ‡ Imperial-X, Imperial College London, London W12 7SL, U.K.

## Abstract

Chalcogenide perovskites such as BaZrS_3_ hold potential as promising photovoltaic materials; however, their integration into solar energy devices is currently limited by the high-temperature processing requirements. To explore alternative low-temperature synthesis pathways, we performed an ab initio thermodynamic analysis, highlighting the critical role of sulfur vapor flux, mainly gaseous S_2_ and S_8_, in driving the synthesis. Our findings reveal that sulfur vapor precursors can provide a thermodynamic driving force 10–10^2^ times stronger than that from traditional solid-state methods. Moreover, we find that sulfur gas composition significantly affects the concentration of sulfur vacancy defects in BaZrS_3_. In particular, for low-temperature synthesis below 600 °C, gaseous S_2_ emerges as the optimal precursor to produce high-quality BaZrS_3_ with reduced defect concentrations. The thermodynamic trend of sulfur vacancy formation is governed by the evaporative nature of sulfur and is independent of specific synthesis reactions. This conclusion holds broader implications for generic chalcogenide synthesis where sulfur vacancy management is important.

## Graphical abstract

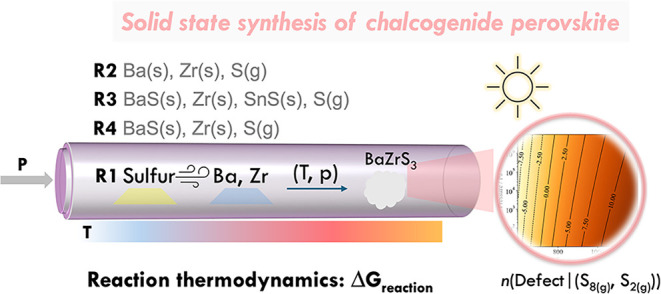

## 1 Introduction

As an emerging chalcogenide-based photovoltaic material, the perovskite phase of BaZrS_3_ has promising photovoltaic properties, such as high photoabsorption coefficient, suitable band gap, and defect tolerance.^1–6^ Like many sulfides, the synthesis of BaZrS_3_ can be tracked back to the 1950s; however, difficulties remained in synthesizing samples that have high crystallinity and uniform film formation.^7^ Due to the brittleness of the ionic structure, even with advanced synthesis techniques today, achieving the high crystallinity growth of BaZrS_3_ film remains difficult, resulting in the predominant study of powder samples.^8–11^ The inhomogeneous grain size and distribution and poor crystallinity of powder samples are detrimental to their photovoltaic efficiency by leading to serious interfacial contact and charge transport issues, severely hindering their application in photovoltaic devices.

In addition, the high synthesis temperature poses another challenge when integrating BaZrS_3_ to photovoltaic devices, due to the high temperature of annealing required to improve the crystalline quality of the materials, which is a process for device fabrication to activate dopants, repair defects, and enhance electrical and optical properties. Thereby, comprehensive growth thermodynamics is essential here to provide insights into directing the design of BaZrS_3_ synthesis conditions.

Another aspect for achieving high-quality samples is the mitigation or reduction of defects. A common defect found in chalcogenides is anion deficiency,^12,13^ attributed to the lower formation energy of V_S_^2+^ compared with other defect species.^14^ This defect formation behavior is also closely tied to the thermodynamic growth conditions, such as the partial pressures (*p*) of gaseous precursors and temperature (*T*), which influence the formation of defect species by altering their chemical potentials. Moreover, the complex sulfur chemistry in the vapor phase would exacerbate the intricacy of this interplay, particularly concerning sulfur-related defects.^15–17^

To address these challenges, we calculate the thermodynamics of BaZrS_3_ growth via various synthesis routes, while also examining their impact on defect formation, particularly in relation to the sulfur chemistry in the vapor phase.

### 1.1 Routes for Synthesizing BaZrS_3_

A range of strategies exist for synthesizing solid-state materials, such as (1) solid-state reactions, usually an elevated temperature is required, (2) gas-phase reactions with flux, (3) hydrothermal reactions, and (4) intercalation or coordination reactions.^15^ The explored synthesis routes to prepare BaZrS_3_ mainly focus on the solid-state and gas-phase reaction strategies,^18^ with a recent progress on using hydrothermal reaction^19^ or in a liquid-assisted way,^20–22^ which are not considered in this work due to the complexity of the solution. In the present work, we only considered gaseous sulfur as the precursor. For reactions involving BaZrO_3_ and H_2_S or CS_2_, the reaction free energies can, in principle, be calculated, as the expected products are BaZrS_3_ and H_2_O or CO_2_. We have confirmed that the necessary thermodynamic data for these gaseous species are available in the NIST-JANAF Thermochemical Tables. This represents an interesting direction for further investigation, which we plan to pursue in our follow-up research. Here, four representative reactions will be discussed, including one solid-state reaction

R1 $$\mathrm{BaS}\left(s\right)+{\mathrm{ZrS}}_{2}\left(s\right)\overset{800-1000\;{}^{\circ}C}{\to }{\mathrm{BaZrS}}_{3}\left(s\right)$$

and three gas-phase reactions.

R2 $$\mathrm{Ba}\left(s\right)+\mathrm{Zr}\left(s\right)+3\;S\left(g\right)\to {\mathrm{BaZrS}}_{3}\left(s\right)$$

R3 $$3\;\mathrm{BaS}\left(s\right)+\mathrm{Zr}\left(s\right)+\mathrm{SnS}\left(s\right)+3\;S\left(g\right)\overset{600\;{}^{\circ}C}{\to }{\mathrm{BaZrS}}_{3}\left(s\right)+{\mathrm{Ba}}_{2}{\mathrm{SnS}}_{4}\left(s\right)$$

R4 $$\mathrm{BaS}\left(s\right)+\mathrm{Zr}\left(s\right)+2\;S\left(g\right)\overset{600\;{}^{\circ}C}{\to }{\mathrm{BaZrS}}_{3}\left(s\right)$$

Some of these reactions have been used for synthesizing chalcogenide perovskites.^4,23,24^ Due to the high chemical reactivity of Ba, Reaction 6 is an elemental reaction that requires meticulous control. In these reactions, high temperatures are usually required. For example, due to the strong stability of BaS and ZrS_2_, the solid-state reactions with metal sulfide binaries necessitate a furnace heated to near 1000 °C;^3^ in contrast, by introducing gaseous sulfur from volatile sulfur precursors, the reaction temperatures could be lowered down to 600 °C.^24^ A recent work focused on the thermodynamic stability of BaZrS_3_, BaS_*x*_, and ZrS_*x*_ using Reactions 5 and 6 found that both reactions were sensitive to sulfur allotropes and the extent of allotrope mixing.^25^ Nevertheless, the full thermodynamic picture necessary to evaluate the BaZrS_3_ synthesis window across the four reactions, as well as the defect formation thermodynamics, remains to be addressed.

## 2 Computational Methods

The thermodynamic analysis required total energies and phonon properties for a range of solids (BaZrS_3_, Ba, Zr, S, BaS, ZrS_2_, SnS, and Ba_2_SnS_4_) and gas phases (S_8_ and S_2_). The associated first-principles calculations were performed using VASP^26,27^ with the standard frozen-core projector augmented-wave method.^28,29^ The cutoff energy for the plane wave basis functions was set to 700 eV. The generalized gradient approximation^30^ of the Perdew–Burke–Ernzerhof functional for solids (PBEsol)^31^ was used for the description of exchange and correlation. We used a 7 × 7 × 7 k-point mesh for structural relaxation until all atoms were relaxed with Hellmann–Feynman forces below 0.01 eV/Å. The role of spin–orbit coupling (SOC) is not considered in this work.

Phonon calculations were computed with the Phonopy^32^ using the finite displacement method to extract the heat capacity and vibrational entropy of the solid phases. The sulfur vacancy defect formation energy (Δ*H*_D,q_) is determined with the Heyd–Scuseria–Ernzerhof (HSE06) hybrid functional^33–35^ again using VASP, and the calculations were conducted following the ShakeNbreak^36,37^ workflow to ensure the ground state configurations of defects. The change of chemical potentials of gaseous S_8_ and S_2_ was treated separately and calculated with PBEsol functional and then combined with Δ*H*_D,q_ to determine the concentration of V_S_^2+^ defect. Full calculation details can be found in our GitHub repository.

## 3 Results and Discussion

### 3.1 Ab Initio Thermodynamics of Synthesizing BaZrS_3_

First-principles calculations offer a powerful tool for accurately predicting the thermodynamics for the synthesis of solid-state materials.^38^ Due to the evaporating nature of sulfur, the growth temperature and its partial pressure will influence the thermodynamic driving force for these synthesis reactions. However, energy minimization with density functional theory (DFT) is based solely on the internal energy and does not account directly for any lattice vibrations or the effect of gaseous pressure.^39,40^

Under the assumption of ideal materials, for species *i* involved in the reaction, by adding its zero-point vibrational energy *E*_*i*_^ZP^ to the DFT computed energy *E*_*i*_^DFT^, we can obtain its Gibbs free energy at zero temperature and zero pressure (*G*_*i*_^0^), and per unit *G*_*i*_^0^ is thermodynamically equivalent to its chemical potential μ_*i*_^0^. Hence, we have μ_*i*_^0^ = *G*_*i*_^0^ = *E*_*i*_^DFT^ + *E*_*i*_^ZP^. For species *i* at given reaction conditions, the chemical potential is μ_*i*_(*T*, *p*) defined by eq 5, where μ_*i*_^θ^ is the reference chemical potential under standard conditions of 298.15 K and 1 bar. [*H*_*i*_^θ^ – *H*_*i*_^0^] is the standard enthalpy and (TS)^θ^ = 298.15 K × S^θ^ is available from the literature data.^39,41^

1 $$\mu_{i}\left(T,p\right)=E_{i}^{\mathrm{DFT}}+E_{i}^{\mathrm{ZP}}+\left[\mu_{i}\left(T,p\right)-\mu_{i}^{\theta }\right]+\left[H_{i}^{\theta }-H_{i}^{0}\right]-{\left(\mathrm{TS}\right)}^{\theta }$$

For the incompressible solid phase, due to the relatively high bulk modulus of BaZrS_3_ (75 GPa),^42^ the influence of pressure and thermal expansion is neglected. Under an absolute pressure *P*, the chemical potential is calculated by

2 $$\mu_{i}\left(T,p_{i}\right)=E^{\mathrm{DFT}}+E^{\mathrm{ZP}}+\left[H_{i}^{\theta }-H_{i}^{0}\right]+\int_{T^{\theta }}^{T}C_{v}\mathrm{dT}+\mathrm{PV}-\mathrm{TS}\left(T,p_{i}^{\theta }\right)$$

while for ideal gases,

3 $$\mu_{i}\left(T,p_{i}\right)=E^{\mathrm{DFT}}+E^{\mathrm{ZP}}+\left[H_{i}^{\theta }-H_{i}^{0}\right]+\int_{T^{\theta }}^{T}C_{p}\mathrm{dT}+\mathrm{RT}\;\ln\left[p_{i}/p_{i}^{\theta }\right]-\mathrm{TS}\left(T,p_{i}^{\theta }\right)$$

Aside from the incompressible solid and ideal gas assumption, the calculation of μ_*i*_(*T*, *p*) requires some data from literature and lattice dynamics calculations. Finally, the reaction free energy Δ*G*_f_ could be calculated from eq 8, where a negative sign of Δ*G*_f_ denotes the reaction toward the product end, and the more negative the value, the higher the driving force for the reaction to occur.

4 $$\Delta G_{f}=\Delta \mu =\sum_{i}\mu_{\mathrm{products},i}-\sum_{i}\mu_{\mathrm{reactants},i}$$

In Figure 1, the reaction free energies (Δ*G*_f_) of synthesizing BaZrS_3_ via Reactions 5–8 under operating window with temperature ranging from 0 to 1000 K and pressure from 0 to 10^7^ Pa are presented. When treating all of the solids as incompressible and treating S as a solid at all temperatures, all four synthesis routes are favored toward the product end driven by the change of free energies.

**Figure 1:**
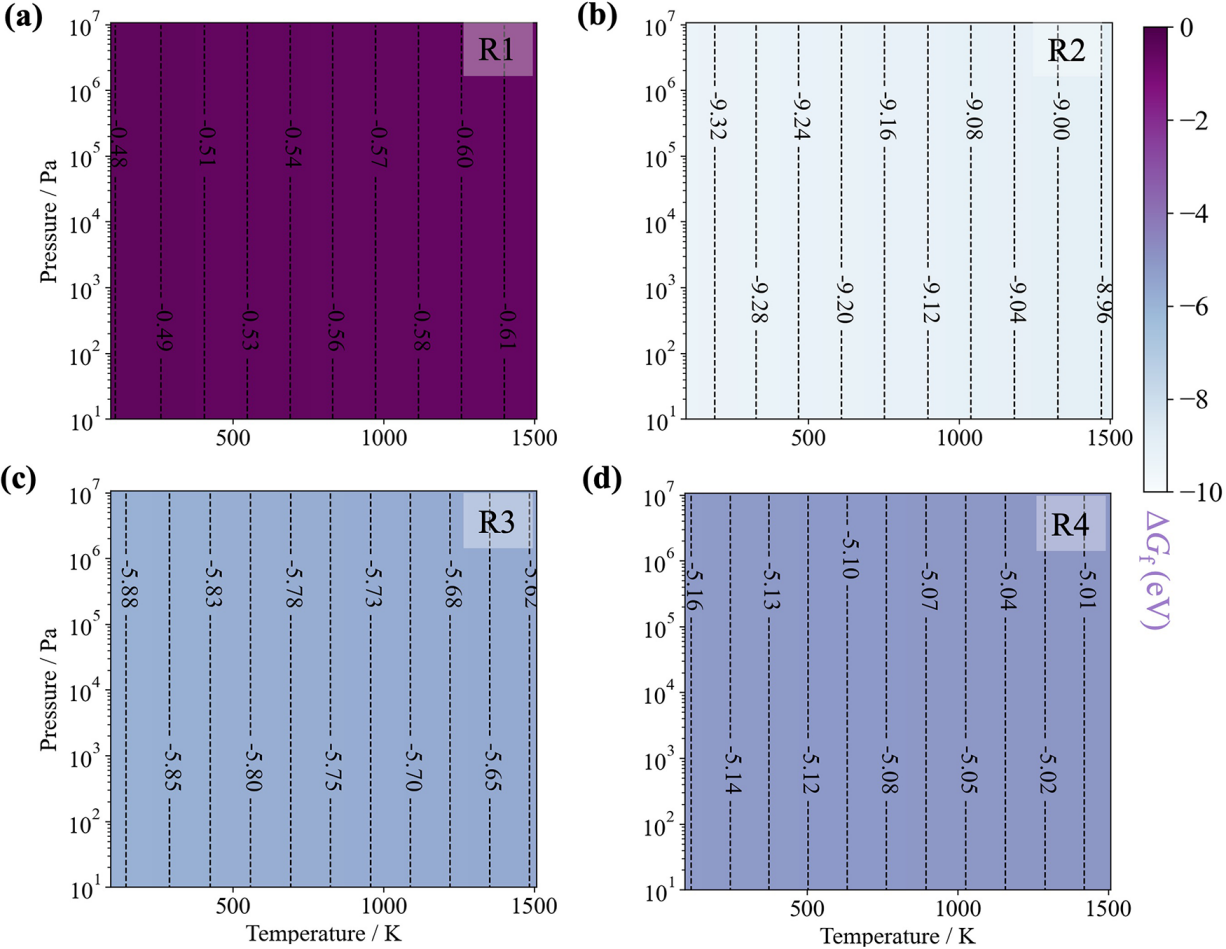
Reaction free energies (eV/f.u.) for synthesizing BaZrS_3_ via Reactions 5–8 (a–d) under an operating window with temperature ranging from 0 to 1000 K and pressure from 0 to 10^7^ Pa, suppose that S remains solid across the entire synthesis window. R1–R4 denote the four reactions.

Reaction 5, initiated with the metal binaries, exhibited the smallest change of Gibbs free energy in the range of −0.48 to −0.60 eV/f.u., indicating a comparatively lower thermodynamic driving force toward product formation. In Reaction 6, the energy change is as much as −9 eV/f.u., reflecting the high reactivity of the elemental substances Ba, Zr, and S. The free energies of Reactions 7 and 8 occupy intermediate positions in the energy spectrum, showing the effect of reactant choice. For instance, in Reaction 7, when modulated with a mixed sulfur source derived from SnS and S, the driving force of reaction was approximately −5.7 eV/f.u. and the reaction temperature was lowered to 600 °C experimentally;^24^ in Reaction 8, employing the inert metal binary BaS and elemental Zr and S as the reactants, the change of free energy is 0.6 eV/f.u. smaller than that in Reaction 7, and reported a similar reaction temperature of 600 °C.^4^ Nonetheless, regardless of the reaction pathways, due to the high stability of BaZrS_3_, the formation of the desired product is favored. Since all reactions are thermodynamically favorable at all temperatures, the temperature is mainly needed to overcome kinetic barriers. Reaction free energies and activation energies can be inversely correlated, so a larger thermodynamic driving force implies a lower kinetic barrier.^43^

In practical experiments, the volatile nature of sulfur will drive the thermodynamic free energy landscape away from all solid-state reactions.^17^ In the gas phase, the evaporated sulfur can manifest as a mixture of S_8_, S_7_, S_6_, and so forth, with their relative mixing ratio fluctuating in response to the growth temperature and sulfur partial pressure, introducing an additional layer of complexity to the thermodynamic evaluation on the synthesis of BaZrS_3_. S_8_ and S_2_ can serve as suitable approximations to the sulfur gas-phase components across the relevant temperature range.^17^ At lower temperatures, bulk sulfur evaporates into the dominating form of S_8_ initially, while as growth temperatures surpass a theoretical 400 °C, S_2_ emerges as the primary form of sulfur vapor. The change in S vapor composition will influence its chemical potential and, in turn, the growth of BaZrS_3_.

The thermodynamic landscapes, with a specific focus on evaporated S_8_ and S_2_ gases across Reactions 6–8 are shown in Figure 2. In the case of gases, the term ∫_*T* ^θ^_^*T*^ *C*_*p*_ dT + RT ln [*p*_*i*_/*p*_*i*_^θ^] in the chemical potential expression (eq 7) dominates the growth driving force. Taking Reaction 6 with elemental substances Ba, Zr, and S as an example, under identical growth conditions (*T*, *p*), if all sulfur was to evaporate as gaseous S_8_, the reaction Δ*G*_f_ was increased by approximately 0.15–1 eV/f.u. across the entire growth window, consequently retarding the driving force for forward reaction. Conversely, if all sulfur was transformed into gaseous S_2_, the reaction would be significantly propelled toward the product end by providing an additional reaction free energy change from 2.7 to 0 eV/f.u. when the growth temperature is increased up to 820 K. Interestingly, with temperature further increasing, the reaction Δ*G*_f_ starts to increase with a magnitude from 0 to 1.8 eV/f.u. across the remaining growth window. This originates from the fact that it crosses the “equivalence” boundary where μ­(α–S) = μ­(S_2_), which will be addressed later.

**Figure 2:**
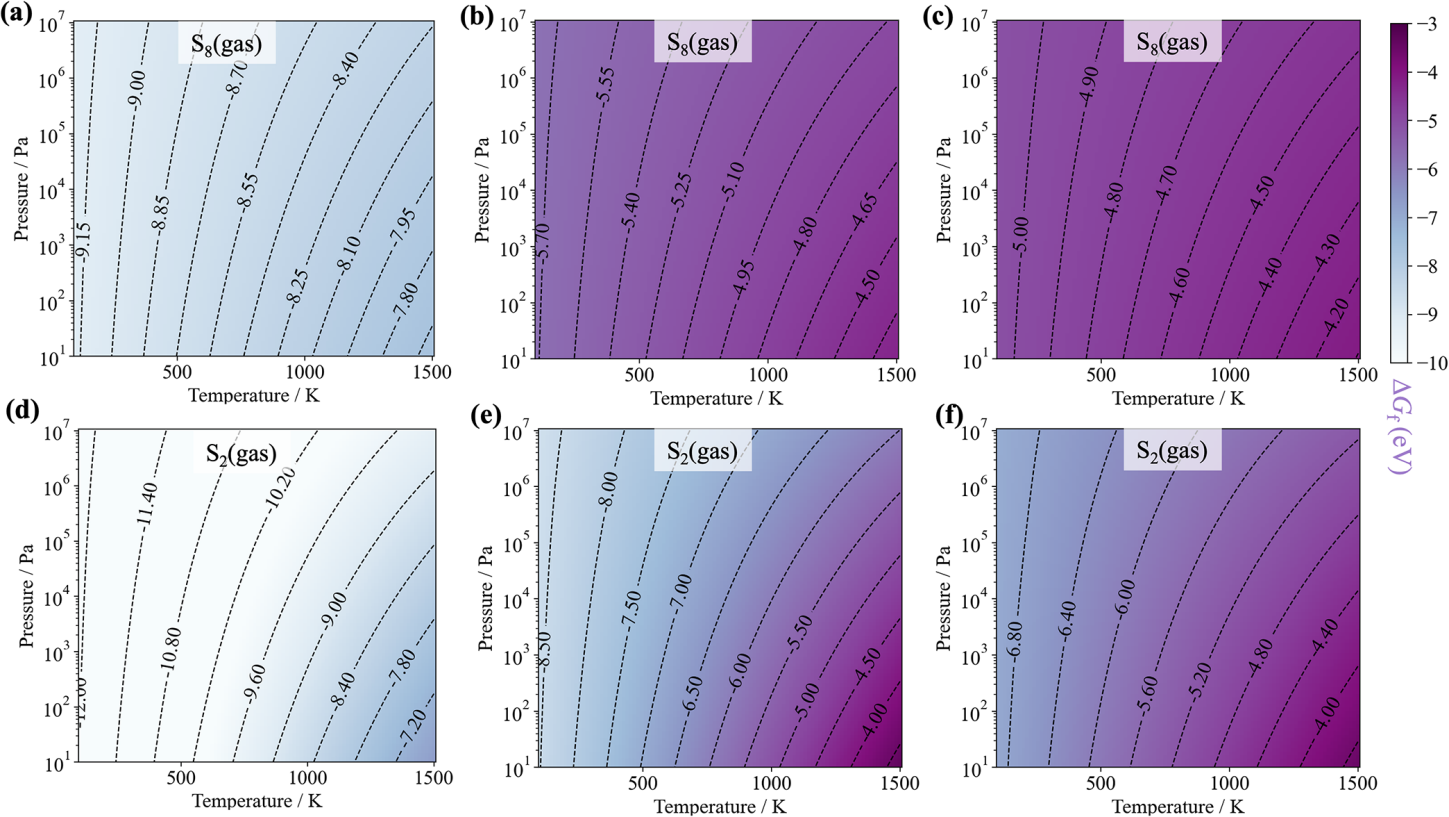
Reaction free energies for synthesizing BaZrS_3_ via Reactions 6–8 considering an operating window spanning temperature from 0 to 1000 K and S partial pressure up to 10^7^ Pa under the evaporated sulfur picture. (a, d) Reaction 6. (b, e) Reaction 7. (c, f) Reaction 8.

Similar trends are observed for Reaction 7 initiated with BaS, Zr, SnS, and S, and Reaction 8 beginning with BaS, Zr, and S. It is worth noting that although SnS is expected to decompose or sublimate across the growth windows, it is treated as an incompressible solid here. Nonetheless, the inclusion of SnS in the reaction stoichiometry plays a crucial role in promoting a more uniform growth of BaZrS_3_,^24^ compared with samples obtained from Reaction 8.^4^ In addition to its role in suppressing oxidation and being thermally removable, the presence of SnS introduces a competing factor for tuning the partial pressure of S in the gas phase, thereby offering a new design strategy for lowering the growth temperatures of BaZrS_3_. Also, as shown in Figure 2, for the thin-film growth of BaZrS_3_ in a vacuum chamber, when the partial pressures of sulfur are much lower, for example, 10^1^ Pa, the reaction still favors the product end thermodynamically while the reaction driving force (Δ*G*_f_) for each reaction temperature will be smaller than that under higher sulfur partial pressure. In this case, temperature plays a critical role in compensating for the loss of reaction driving forces.

In general, the composition of sulfur vapor alters the growth thermodynamics, with gaseous S_8_ generally retarding reactions, while gaseous S_2_ propels them forward. Therefore, the key to achieving a lower temperature synthesis lies in how to prioritize the presence of S_2_ as the primary gaseous component. This can be achieved at elevated temperatures through direct sublimation of α-S or from the breakdown of S_8_ (see Figure S3). We calculated these two reactions following the procedures introduced in ref 17, under the assumption that both α-S and S_8_ can be decomposed into various sulfur species including S_2_. Alternatively, a separate “cracking” stage can be used to feed in activated S_2_ directly as a reactant for synthesis.

### 3.2 Control of V_S_^2+^ Defect Formation

One crucial aspect in growing high-quality BaZrS_3_ for optoelectronic applications is defect control. In BaZrS_3_, there are more than one hundred distinct types of defects that might present, including vacancies, interstitials, and antisites with different charge states (analysis performed via doped^44^). The formation energies for some of these have been reported before without detailed consideration of the growth environments.^3,14^

We focus on the important case of the sulfur vacancy, which has the potential to act as an electron trap and nonradiative recombination center in metal sulfide semiconductors.^45,46^ Ref 46 presents a modeling study based on 0 K DFT simulations that identifies a potential deep transition-level defect–sulfur interstitials. Our work agrees with their conclusion that sulfur vacancies exhibit shallower defect transition levels at 0 K. Furthermore, we extend this understanding by investigating the defect behavior under finite temperature and pressure growth conditions. The formation energy is calculated using the standard formalism for charged defects in solids,

5 $$\Delta H_{D,q}\left(E_{F},\mu \right)=\left[E_{D,q}-E_{H}\right]+\sum_{i}n_{i}\mu_{i}+qE_{F}+E_{\mathrm{corr}}$$

where the electronic and chemical potentials for forming a defect D in charge state q are determined by *E*_F_ and μ, respectively. Corrections for finite-size effects (*E*_corr_) are implemented following the approach of Kumagai and Oba.^47^

As shown in Figure 3b, among V_S_^2+^, V_S_^1+^, V_S_^0^, V_S_^1–^, and V_S_^2–^, V_S_^2+^ has the lowest formation energy. We assume an intrinsic semiconductor where the equilibrium Fermi level is placed in the center of the band gap. Under the S-deficient conditions, the formation energy of V_S_^2+^ is about 1.5 eV (Figure 3b), which can be tuned by the sulfur chemical potentials. Under S-rich conditions, the formation energy is increased to around 3 eV (Figure S2). It is important to note that lower defect formation energy corresponds to higher equilibrium defect concentrations. Therefore, our focus is on manipulating the S-deficient conditions to optimize the defect population.

**Figure 3:**
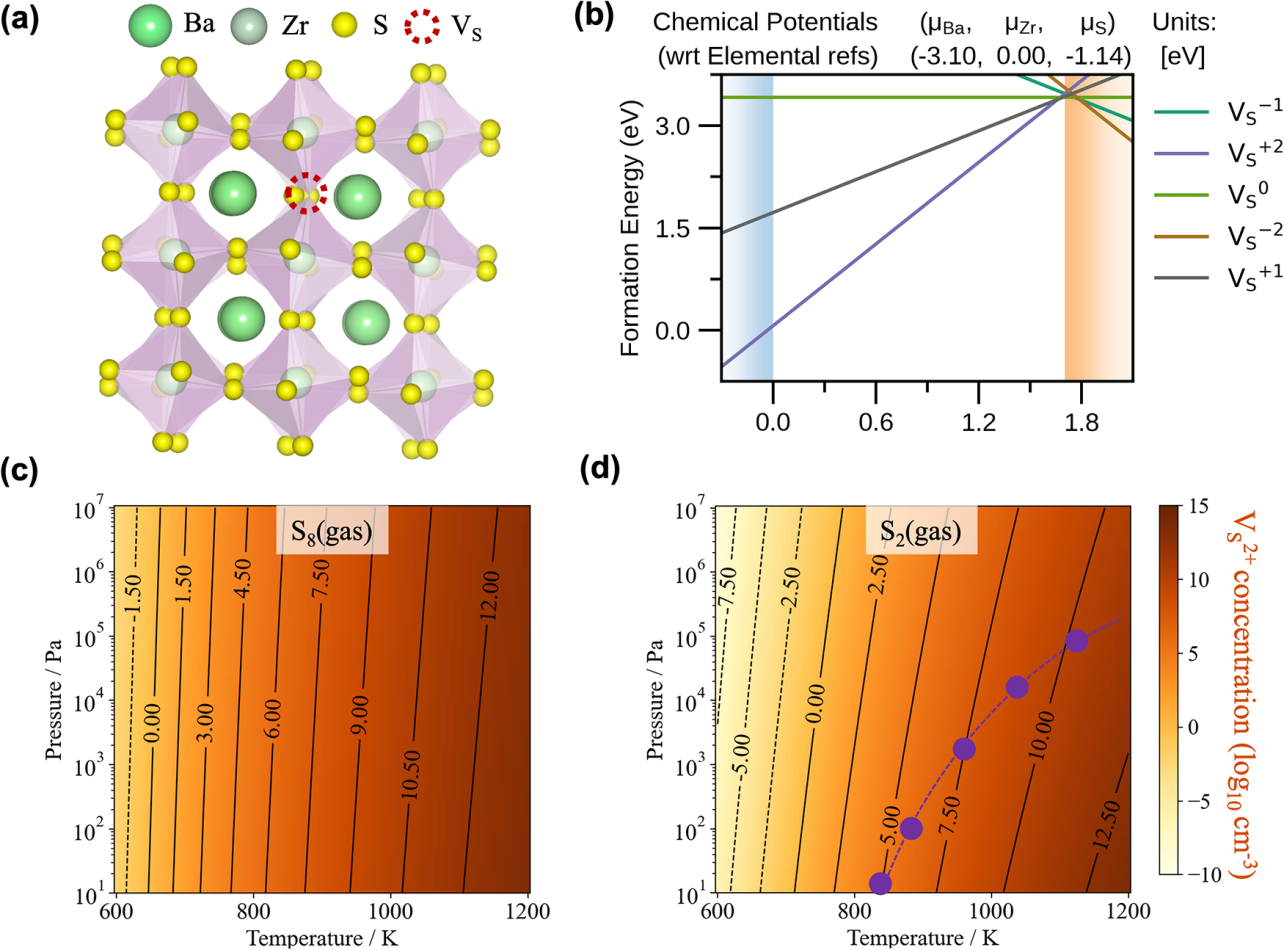
(a) Sulfur vacancy in a BaZrS_3_ crystal and (b) formation energy in five charge states as a function of the Fermi level under S-poor conditions. Predicted defect concentration for equilibrium with respect to (c) S_8_ and (d) S_2_ vapor as a function of temperature and pressure. The purple dashed line on (d) denotes the boundary when the defect concentration is equal using either solid-state α-S(s) or S_2_(g) as the growth precursor.

From Figure 3c,d, we can find that higher growth temperatures consistently result in higher defect concentrations. Pressure poses minimal effect on defect formation in incompressive solid-state reactions when using α-S as the growth precursor with the highest V_S_^2+^ concentration at the level of 10^11^ cm^–3^, while gaseous S_8_ leads to higher defect concentrations across the entire growth window compared with reactions with α-S, showing the highest V_S_^2+^ concentration over 10^12^ cm^–3^. Meanwhile, using pure gaseous S_2_ as the growth precursor, as shown in Figure 3d, the boundary is delineated denoting the growth conditions where the defect concentration is equivalent when using either solid-state α-S or S_2_ gas as the growth precursors. To the right side of the boundary, where the growth temperature exceeds 820 K and the pressure is below 1 bar, solid-state α-S is preferred for reducing the defect concentration. However, under most of the growth windows below 820 K, gaseous S_2_ proves favorable for growing high-quality crystals, with defect concentration being substantially lower than using other sulfur sources.

It is worth noting that the absolute defect concentrations of V_S_^2+^ in BaZrS_3_ under all conditions are low because of the high defect formation energy. However, the comparative orders of magnitude change in defect concentration are significant. A reduction of one to two orders in defect concentration can have a profound impact on material properties. This influence is general and arises from the difference in chemical potential

6 $$\Delta \mu_{i}\left(T,p_{i}\right)\left(S_{s}\to S_{g}\right)=\mu_{i}(T,p_{i}{)}_{\left(S_{g}\right)}-\mu_{i}(T,p_{i}{)}_{\left(S_{s}\right)}$$

which changes the V_S_ formation energy (eq 9).

In consensus with other modeling and experimental works,^3,14,46,48^ BaZrS_3_ is a defect-tolerant material for efficient solar energy conversion, with shallow defect transition levels for almost all types of vacancies, interstitials, and antisite defects; our results also agree with the experimental observation that as-grown BaZrS_3_ is intrinsically *n*-type, due to the ease of formation of sulfur vacancy. Also recently presented in ref 46, under the S-rich condition, sulfur interstitials can be potential nonradiative recombination centers in this material.

The density of V_S_ defects demonstrates a correlation with the annealing temperature and duration. This observation falls into a family of thermodynamic relations governing defect formation with gaseous precursors, such as O_2_ used in metal oxide synthesis, where the formation energies of intrinsic point defects are strongly coupled to the partial pressure of oxygen.^49,50^

## 4 Conclusions

Through our ab initio thermodynamic calculations on different BaZrS_3_ synthesis strategies, the reaction Gibbs free energy results reveal that synthesis is favorable over a large temperature and pressure range. This outcome persists even when initiating the reaction with stable metal binaries BaS and ZrS_2_ as the growth precursors. Therefore, the feasibility of low-temperature synthesis is primarily constrained by the reactivity of precursors. In the context of gas flux-assisted synthesis, we have highlighted that S_2_ proves beneficial for growing high-quality samples compared with other states of sulfur. This also carries through to the control of equilibrium sulfur vacancy concentrations. Additionally, there is a recent trend in liquid-assisted growth of BaZrS_3_ to further lower the reaction temperatures, which we perceive to be somewhat analogous to the gas flux-assisted synthesis, albeit more complex due to the difficulty in describing the activity of reaction precursors in the solution.

## Supplementary Material



## Notes

Figure S1: Chemical potential and phase diagram of Ba, Zr, S; Figure S2: Sulfur vacancy formation energy as a function of the Fermi level under different sulfur chemical potentials. Figure S3: Reaction free energies for alpha sulfur evaporating into the gaseous (a) S_8_, (c) S_2_, and (b) gaseous S_8_ transforming to S_2_ (PDF)
