# Supplementary material for: Thermodynamics of Sulfur Vacancy Formation in the Chalcogenide Perovskite BaZrS3
Source: J Phys Chem C Nanomater Interfaces. 2025 Oct 22;129(43):19473–9. doi: 10.1021/acs.jpcc.5c00828 (PMC12581149; doi:10.1021/acs.jpcc.5c00828)
Supplement: Supplementary file 1 [file jp5c00828_si_002.pdf]

Supporting Information: Thermodynamics of Sulfur Vacancy Formation in the Chalcogenide Perovskite $BaZrS_3$

Zhenzhu Li*,†,‡ and Aron Walsh†,¶

†*Department of Materials, Imperial College London, London SW7 2AZ, UK*

‡*Imperial-X, Imperial College London, W12 7SL, UK*

¶*Department of Physics, Ewha Womans University, Seoul 03760, Korea*

E-mail: zhenzhu.li@imperial.ac.uk

Phase diagram of Ba, Zr, and S.

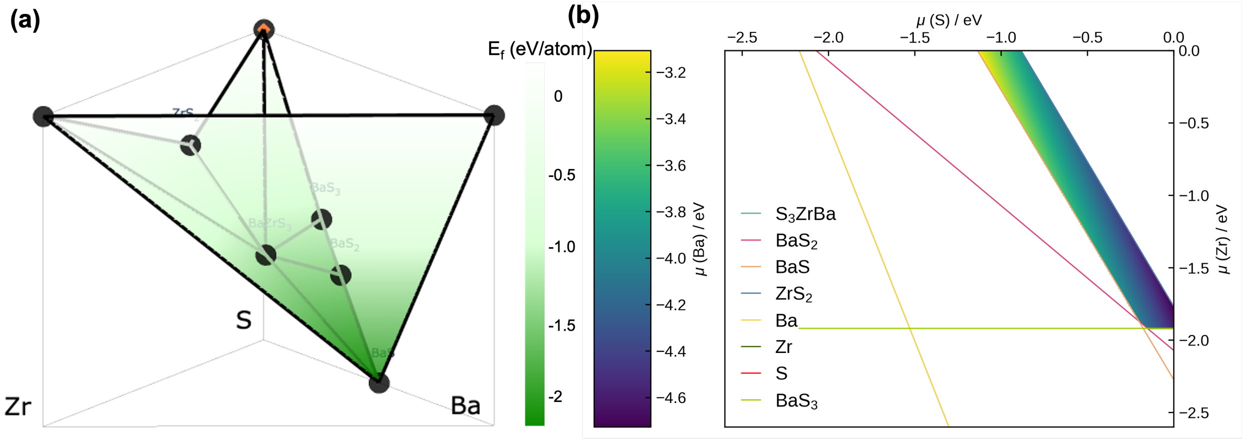

Figure S1: Chemical potential and phase diagram of Ba, Zr, S.

The phase diagram of Ba, Zr, and S was calculated with HSE06 functionals by considering the possible decomposition pathways of $BaZrS_3$ into binary and elementary phases. Other

ternary phases such as $Ba_3Zr_2S_7$, Ba_2ZrS_4 and $Ba_4Zr_3S_{10}$ were not considered. The obtained chemical potential range was used for the calculation of defect formation windows.

S vacancy formation energies under S poor/rich conditions.

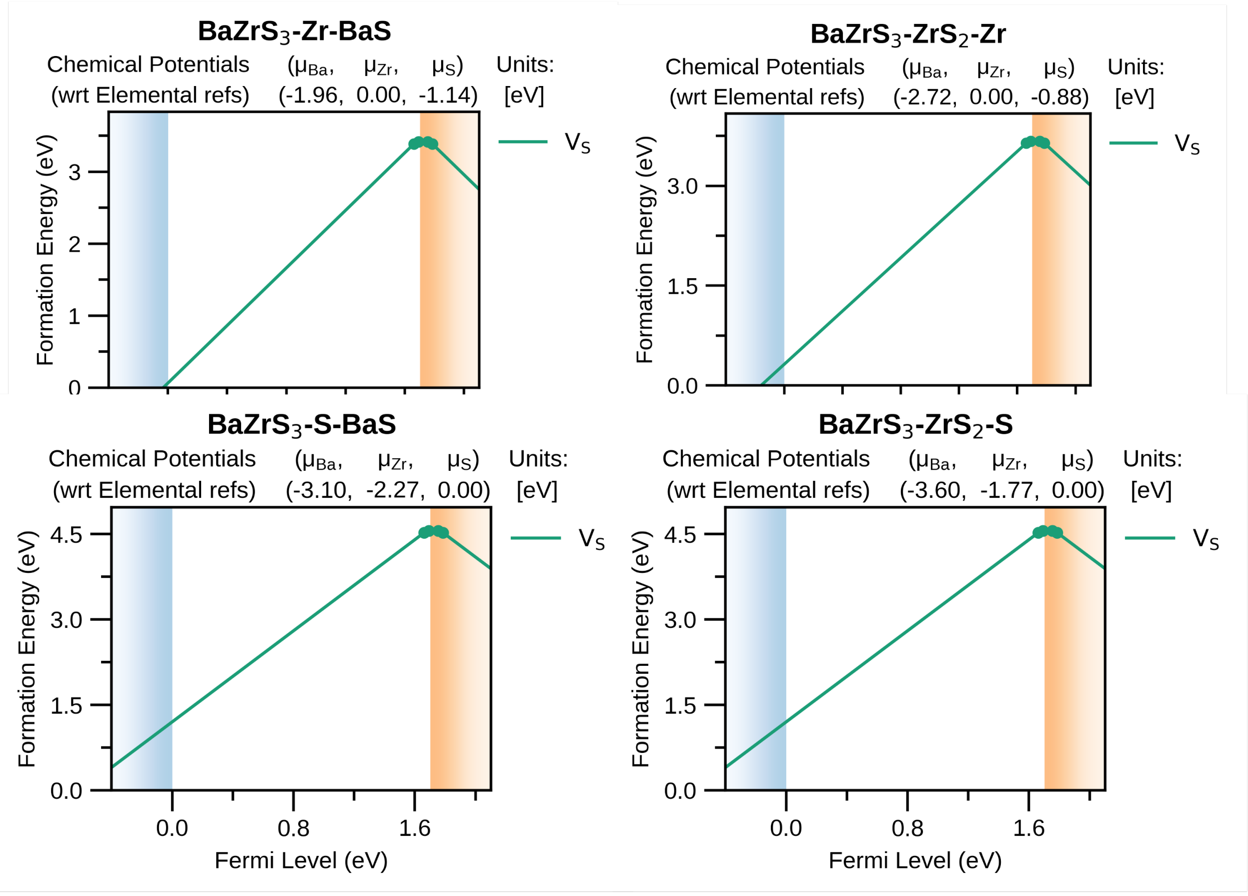

Figure S2: Sulfur vacancy formation energy as a function of the Fermi level under different sulfur chemical potentials.

All the defect configurations were calculated with the HSE06 functional. For charged defects, the correction for finite-size effects (E_{corr}) were implemented following the approach of Kumagai and Oba. In total, 127 defects including interstitials, vacancies, and anti-sites were generated for our defect caculations, for this work, only sulfur vacancies were considered.

The overall defect formation energy of sulfur vacancy is high in the $BaZrS_3$ material and showing a shallow transition levels, indicating a benign nature of this type of defect. The difference of vacancy formation energy under sulfur poor and sulfur rich conditions is about 1eV.

Transitions among sulfur allotropes.

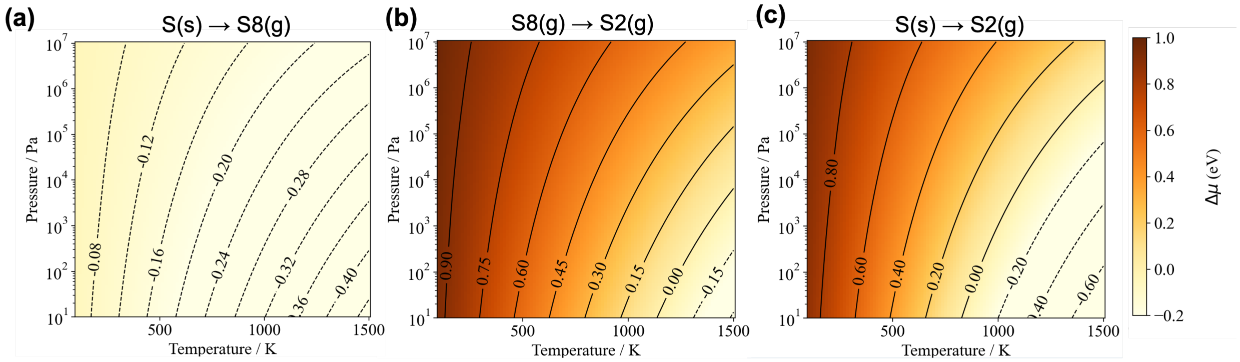

Figure S3: Reaction free energies for alpha sulfur evaporating into the gaseous (a) S_8, (c) S_2, and (b) gaseous S_8 transforming to S_2.

We calculated that the formation of gaseous S_2 could both originate from the sublimation of α-sulfur and the decomposition of gaseous S_8 at elevated temperatures. Figure S3a shows that α-sulfur can spontaneously sublime into the gaseous S_8 across the entire temperature and pressure range, while the transformation into gaseous S_2 occurs after crossing the equivalent chemical potential boundary where $\mu(\alpha - \mathrm{S}) = \mu(\mathrm{S}_2)$ (T=820 K, the $\Delta\mu$=0.00 line in Figure S3c). In contrast, Figure S3b indicates that the gas phase decomposition of S_8 into S_2 necessitates approximately 300 K higher temperature to reach the equivalent chemical potential boundary where $\mu(\mathrm{S}_8) = \mu(\mathrm{S}_2)$ (T=1100 K, the $\Delta\mu$=0.00 line in Figure S3b). This suggests that prior to the decomposition of gaseous S_8, the source of gaseous S_2 primarily comes from the sublimation of α-sulfur to facilitate the low temperature utilization of S_2, while in real application, we suggest to set up a separate chamber to be used for the generation of gaseous S_2.
